# Mindfulness-Based Interventions for Smoking Cessation in People with cancer: Insights from Qualitative Data

**DOI:** 10.21203/rs.3.rs-10856341/v1

**Published:** 2026-10-14

**Authors:** Natalia Van Doren, Kylie Sloan, Edgar Yu, Carmen Ma, Chigozie Onyeagoro, Mei-Chin Kuo, Zinnia Loya, Laurel Bray-Hannin, Stella Bialous, Chloe Atreya, Patrick Ha, Lisa Kroon, Sue Yom, Janice Tsoh

## Abstract

Background Continued tobacco use after a cancer diagnosis substantially worsens treatment outcomes and survival; yet, over 60% of people with cancer continue to smoke and fewer than 30% receive cessation support. Standard cessation approaches may fail to address the unique psychological context of cancer care, which includes cancer-related distress, perceived loss of autonomy, and heightened stigma. Mindfulness-based interventions (MBIs) are theoretically well-suited to address these mechanisms, yet have rarely been adapted to target the specific barriers to cessation in oncology settings. Method A subset of participants enrolled in CareConnect, a randomized clinical trial of tailored digital outreach for tobacco cessation in a racially and ethnically diverse sample of adult people with cancer who had cancer treatment within the past three months and completed semi-structured interviews ( *N* = 24), 3 months to 1 year after receiving the outreach call to assess their experiences with the intervention. Thematic analysis was used to identify themes related to engagement with cessation support and how to apply these themes to mindfulness-based interventions. Results Thematic analysis revealed three primary themes maintaining tobacco use and limiting engagement with cessation: (1) autonomy threat and perceived judgment in cancer care; (2) cancer-related distress and treatment overload; and (3) shame, identity threat, and moralization of smoking. Together, these mechanisms constitute a reinforcing the nested Smoking-Distress-Avoidance Cycle in Cancer Care. Conclusions Findings are used to specify MBI components that map onto identified intervention targets. Implications for developing mindfulness-based interventions for individuals with cancer who smoke are discussed.

